# Relation Between Temperament and School Adjustment in Spanish Children: A Person-Centered Approach

**DOI:** 10.3389/fpsyg.2020.00250

**Published:** 2020-02-19

**Authors:** Ester Ato, María Ángeles Fernández-Vilar, María Dolores Galián

**Affiliations:** Department of Developmental Psychology, University of Murcia, Murcia, Spain

**Keywords:** temperament, academic achievement, social adjustment, person-centered approach, latent profile analyses

## Abstract

The aim of this study was to examine from a person-centered approach the impact of temperament on academic achievement and sociometric status in a sample of 6–7-year-old Spanish children. To measure children’s temperament in early childhood, parents were given TMCQ (Temperament in Middle Childhood Questionnaire), while sociometric status and academic achievement were requested for children’s teachers. Using latent profile analysis (LPA) four temperament profiles were found. Children belonged to the “Negative/Undercontrolled” profile showed a higher probability of academic failure and were more rejected, and children included in the profile “Sociable/High regulated” showed higher academic scores and a lower probability of being rejected by their peers. Several implications in the Spanish educational context are discussed.

## Introduction

Academic failure and social maladaptation constitute distressful and ongoing problems in Spain. Considered by recent Ministers of Education as the main problem in Spanish education, school dropout has increased alarmingly in recent years, and is the second highest in the EU (Ministry of Education and training, 2019). Indeed, the latest available data from the PISA report (Programme for International Student Assessment) highlight Spain’s poor performance, with scores below average in both Language and Mathematics (INEE, 2015), compared with other countries. Furthermore, disorders related to social maladjustment in schools, like bullying, anxiety and depression, have also been increased considerably. Concretely, bullying scaled up almost 50% from 2015 to 2017, exceeding for the first time 1000 victims annually in Spain (Report Bullying sin fronteras, 2018). In the search for the most important contributors to explain academic and social outcomes, children’s temperament has emerged strongly, based on the idea that children’s individual differences in emotional arousal could be a protective or a risk factor in their developmental trajectories (Leve et al., 2005).

Temperament has been defined as individual differences in reactivity and self-regulation influenced by maturity and experience (Rothbart, 1981), and its correlation with academic and social adjustment are well established. However, not all the dimensions of temperament contribute equally in explaining children’s adjustment. Specifically, high levels of negative emotionality and low levels of effortful control, defined as the ability to suppress a dominant response to perform a subdominant response (Rothbart and Bates, 1998), have been significantly related to poor academic achievement (Blair and Razza, 2007; Valiente et al., 2008; Zhou et al., 2010; Hintsanen et al., 2012) and worse social adjustment (Eisenberg et al., 2000), while the contribution of positive emotionality is not so clear. Nevertheless, although these relations have been systematically reported in the literature, there are few recent studies relating these variables in Spain (Checa and Rueda, 2011; Ato et al., 2014; Checa and Abundis-Gutierrez, 2017; Galián et al., 2018). Thus, and similar to the results found in other cultures, self-regulation abilities have been proved to have a linear or indirect effect on academic achievement or social maladjustment. Particularly, individual differences in effortful control predicted academic achievement (Checa and Rueda, 2011; Checa and Abundis-Gutierrez, 2017; Galián et al., 2018), and sociometric status in Spanish children (Ato et al., 2014; Sánchez-Pérez et al., 2015) so less self-regulated children were those with lower academic performance and a higher probability of being rejected.

Nevertheless, these relations have been examined mainly thorough a variable-centered approach, which treats dimensions (or overarching factors) as independent entities that uniquely predict their relations with other outcomes. This approach, although useful, is incomplete as it tends to separate psychological processes from the individual in whom they occur and ignores the organization of multiple traits within an individual (Hart et al., 2003). To the extent that this approach lacks of a multilevel consideration of the problem, it seems to be inadequate when the objective is to establish conclusions about individuals (Crockett et al., 2006). In contrast, the person-centered approach is holistic, and the individual is viewed as the unit of analysis and each trait takes on meaning based upon its role within the entire organization of the individual (Bergman and Magnusson, 1997). Thus, from this perspective it is possible to examine how unique combinations of temperament dimensions act together in predicting child outcomes.

In the temperament field, Thomas, Chess and Birch’s pioneer work (1968), based on a person-centered approach, distinguished three children temperament profiles; difficult, easy and slow to warm up, related to socioemotional adjustment, with the difficult temperament outweighing compared to the other two for greater risk of maladjustment (Giancola et al., 1998; Moffitt et al., 2001; Frick and Morris, 2004). In this vein, McClowry (2002) identified four temperament profiles, of which “Social/Eager to try” and “Industrious” would be similar to the Easy profile, “High Maintenance” would be similar to the Difficult profile, and “Cautious/slow to warm up” would be similar to the Slow to warm up profile. Another classic study on profiles is Caspi and Silva (1995), who identified five temperament profiles and examined them in a longitudinal study from 3 to 18 years old. The five categories - undercontrolled, inhibited, confident, reserved and well-adjusted - showed striking differences in adjustment scores, with the undercontrolled being those who showed the worst adjustment in adulthood. More recently, Sanson et al. (2009) identified four temperament profiles which they called reactive/inhibited, poor attention regulation, non-reactive/outgoing and high attention regulation. In their work, children assigned to the reactive/inhibited and poor attention regulation groups tended to have higher levels of later behavior problems compared to children assigned to non-reactive/outgoing and high attention regulation categories, confirming that belonging to one or another profile has a strong impact on their present and future adjustment. Nevertheless, to date there are few studies exploring the effect of temperament on academic achievement and sociometric status from a person-centered perspective, and we have found no such studies for Spain that incorporate both academic and social outcomes.

Based on the aforementioned, the aims in this study were to determine, using Latent Profile Analysis (LPA), temperament profiles in Spanish children aged 6 and 7 years, and to analyze their impact on Math and Language achievement, on the one hand, and to Acceptance and Rejection scores, on the other. Taking into account the luck of studies in the field, this study hopefully will contribute to a better understanding of the variables involved in academic failure and social rejection, which is an important issue that needs to be addressed, particularly in Spain. Besides, we consider the start of childhood a crucial period in the analysis of these relations, insofar as this is the developmental stage in which we usually observe first academic and social problems in the school.

## Materials and Methods

### Participants

This research used data from a larger study of child temperament and its relations with their adjustment in several areas. The participating families were recruited from five schools in Murcia (Spain). Although the larger sample of our research included 474 children from the First and Second Cycle of Primary School, for the purposes of our study we selected only First Cycle of Primary School children. The sample comprised 295 Spanish children (51.2% boys and 48.8% girls) of 6 (44.4%), and 7 (55.6%) years old. The most important reason behind this choice was the consideration that change from first to second cycle could imply academic variations which could alter the relations between temperament profiles and academic performance. Also, as we have just mentioned, we were particularly interested in studying these relations in the start of childhood. Of the participating families, 9.3% of the parents had completed Primary School studies, 22.1% had completed Secondary School, 29.3% held a professional qualification, 28.3% were university graduates, and 1% held a Ph.D.

### Measures

#### Temperament

Temperament was measured using the Temperament in Middle Childhood Questionnaire (TMCQ; Simonds and Rothbart, 2004). This questionnaire obtains information provided by parents on a number of daily situations and includes 160 items on a 5-point Likert scale grouped in 17 temperament scales: (T1) *Activation Control*: The capacity to perform an action when there is a strong tendency to avoid it; (T2) *Activity level***:** The level of gross motor activity including the rate and extent of locomotion; (T3)*Affiliation:* The desire for warmth and closeness with others, independent of shyness or extraversion; (T4) *Anger/frustration*: The amount of negative effect related to interruption of ongoing tasks or goal blocking; (T5) *Assertiveness/dominance*: The tendency to speak without hesitation and to gain and maintain control of social situations; (T6) *Attentional Focusing*: The tendency to maintain attentional focus upon task-related channels; (T7) *Discomfort:* The amount of negative effect related to sensory qualities of stimulation, including the intensity, rate or complexity of light, movement, sound and texture; (T8) *Fantasy/Openness:* Active imagination, esthetic sensitivity and intellectual curiosity; (T9) *Fear:* The amount of negative affect including unease, worry or nervousness related to anticipated pain or distress and/or potentially threatening situations; (T10) *High Intensity Pleasure:* The amount of pleasure or enjoyment related to situations involving high stimulus intensity, rate, complexity, novelty and incongruity; (T11) *Impulsivity*: The speed of response initiation; (T12) *Inhibitory Control:* The capacity to plan and to suppress inappropriate approach responses under instructions or in novel or uncertain situations; (T13) *Low Intensity Pleasure:* The amount of pleasure or enjoyment related to situations involving low stimulus intensity, rate, complexity, novelty and incongruity; (T14) *Perceptual Sensitivity*: The amount of detection of slight, low intensity stimuli from the external environment; (T15) *Sadness*: The amount of negative affect and lowered mood and energy related to exposure to suffering, disappointment and object loss; (T16) *Shyness:* Slow of inhibited approach in situations involving novelty or uncertainty; and (T17) *Soothability/Falling Reactivity:* The rate of recovery from peak distress, excitement or general arousal. The coefficient alpha for these subscales ranged from 0.621 to 0.887.

#### Sociometric Status

Sociometric status data were collected using the BULL-S Questionnaire (Cerezo, 2000), a questionnaire designed to measure, among other aspects, the sociometric position of each person of the group. For that purpose, the teachers administered a sociogram in which the children answered, in order of preference, about three other children with whom they least and most liked (1) working, and (2) spending their free time (in the classroom context). After that, for each of the participating children a rejection score (RS) and an acceptance score (AS) was calculated following the procedure detailed in the reference manual (Cerezo, 2000, p. 28–31).

#### Academic Performance

At the end of the academic year the students’ final grades in Language (LP) and Math (MP) were recorded. The scores provided by the teachers were distributed in 4 categories (Fail, Pass, Merit, and Distinction). We categorized the variable from Fail = 1 to Distinction = 4.

### Procedure

A meeting was held with the head teachers of the schools in order to explain the purpose of the project to them. After consent was given by the school and parents, a second meeting was held with the tutors to instruct them in the administration of the sociometric test. At the same time, they were given the temperament questionnaire, along with a letter addressed to the parents with instructions for filling in it. A telephone number for queries was also provided. When the tests were filled in, a third meeting with tutors and parents was held to solve or correct possible mistakes detected in the questionnaires. Questionnaires that were less than 80% completed were discarded.

### Data Analysis

Complete data were collected for all 295 cases of the sample. No missing data were found. We first used a descriptive analysis of all the variables measured and ANOVA tests for gender differences. Then we ran a Latent Profile Analysis (LPA) to determine children’s temperament profiles with the *tidyLPA* (version 1.0.2) package of *R* platform (Rosenberg et al., 2018), interfaced with *MPlus* (version 8.1, Muthén and Muthén, 1998–2017) via *MPlusAutomationR* program (Hallquist and Wiley, 2018). LPA is a special case of the general mixture model in which latent profiles are identified based on patterns of observed indicator variables. Latent profiles differ from latent classes due to the continuous nature of measured variables (Harring and Hodis, 2016). In our study, LPA is used mainly to discern the optimal covariance matrix and the number of children’s subsets (profiles) who share similar patterns of temperament attributes.

In order to help in the interpretation of profiles we also used Partial Least Squares (PLS) regression, a dimension reduction technique where predictor variables are mapped onto a smaller set of variables that maximally explain a response variable. The package *pls* of R platform (version 2.7, Mevik et al., 2019) was selected, instead of linear regression analysis, to avoid multicollinearity problems and to enable the set of the 17 temperament scales to be ranked based upon how strongly they influence on each of the 4 profiles obtained by LPA. Finally, we also used planned comparisons ANOVA to detect plausible profile differences and gender differences in social and academic adjustment measures.

## Results

### Descriptive Statistics, Correlations and Gender Differences

Table 1 shows descriptive data and significant correlations between the variables of interest. ANOVA tests were applied to check for statistically significant differences in gender and age for each of the measures. Significant differences between boys and girls were found for T4: *F*(1,264) = 3.84, *p* = 0.05; T6: *F*(1,264) = 23.17, *p* < 0.001; T7: *F*(1,264) = 6.71, *p* = 0.01; T8: *F*(1,264) = 25.60, *p* < 0.001; T11: *F*(1,264) = 6.57, *p* = 0.01; T12: *F*(1,264) = 13.96, *p* < 0.001; T13: *F*(1,264) = 6.61, *p* = 0.01; T14: *F*(1,264) = 7.81, *p* < 0.01; Rejection: *F*(1,264) = 14.20, *p* < 0.001 and Language achievement: *F*(1,264) = 8.05, *p* < 0.01, with boys scoring higher in T4, T7, T11, and Rejection, whereas girls scored higher in T6, T8, T12, T13, T14, and Language achievement.

**TABLE 1:** Descriptive data and significant correlations.

	T1	T2	T3	T4	T5	T6	T7	T8	T9	T10	T11	T12	T13	T14	T15	T16	T17	AS	RS	LP	MP
Mean	3.34	4.09	4.24	3.19	3.43	3.24	2.86	3.97	2.89	3.30	2.98	3.20	3.98	3.70	2.66	2.78	3.48	6.50	5.11	3.25	3.25
SD	0.50	0.61	0.43	0.72	0.54	0.96	0.61	0.54	0.61	0.64	0.67	0.57	0.50	0.60	0.59	0.96	0.59	5.16	7.29	0.77	0.80
AS						0.3***				−0.1*	−0.2***	0.2**	0.1**						−0.4***	0.3***	0.3***
RS	−0.1*	−0.1*		0.1*		−0.4***	−0.4***	−0.1*			0.3***	−0.2***	−0.2***				−0.1*	−0.4***		−0.4***	−0.3***
LP	0.1*					0.4***		0.1*		−0.1*	−0.2*	0.1*	0.1*					0.3***	−0.4***		0.8***
MP						0.4***					0.1*	0.1*						0.3***	−0.3***	0.8***	

T1, Activation control; T2, Activity level; T3, Affiliation; T4, Anger/Frustration; T5, Assertiveness/Dominance; T6, Attentional Focusing; T7, Discomfort; T8, Fantasy/Openness; T9, Fear; T10, High Intensity Pleasure; T11, Impulsivity; T12, Inhibitory Control; T13, Low Intensity Pleasure; T14, Perceptual Sensitivity; T15, Sadness; T16 Shyness; T17, Soothability/Falling Reactivity; AS, Acceptance Scoring; RS, Rejection Scoring; LP, Language Performance; MP, Math Performance.*p < 0.05; **p < 0.01; ***p < 0.001.

No significant differences between children of 6 and 7 years old were found in any of the social and academic performance temperament measures.

### Latent Profile Analysis and Partial Least Squares Regression

The 17 temperament scales of TMCQ were then subjected to a latent profile analysis (LPA) in order to select the optimum number of profiles. LPA is a model-based clustering technique that provides a precise framework for choosing a relevant number of clusters/profiles with the most appropriate covariance matrix. A first look at the covariance matrix of all temperament measures showed variances in the range 0.25/0.92 and covariances in the range -0.32/ + 0.29. Then we focused our attention on models whose covariance structures show equal or varying variances and zero covariances. These structures correspond to models 1 and 2 of the *tidyLPA* R-package (Rosenberg et al., 2019) and were estimated using the most popular range between 2 and 5 profiles. Table 2 shows the most relevant information criteria used to choose the best combination of covariance structure and the number of profiles (lower values are better). Classical Akaike Information Criterion (AIC) and Bayesian Information Criterion (BIC) give contradictory information (AIC for model 2 with 5 profiles; BIC for model 1 with 4 profiles). The adjusted sample size BIC (SABIC; Sclove, 1987) pointed again toward model 1 with 5 profiles, but with the most robust Integrated Completed Likelihood (ICL, Biernacki et al., 1998; Baudry, 2015), the best fit model would be a 4-profile model with a covariance structure assuming equal variances and zero covariances.

**TABLE 2:** Selection of the covariance matrix and number of profiles.

Covariance Structure	Profiles	AIC	BIC	SABIC	ICL
Model 1: Equal	2	9090	8282	9117	9319
variances,	3	8886	9144	8922	9206
Zero covariances	4	8711	9035	8756	9108
	5	8595	8986	8649	9131
Model 2: Varying	2	9076	9330	9112	9368
variances,	3	8857	9240	8910	9280
Zero covariances	4	8661	9174	8732	9224
	5	8576	9218	8666	9269

**FIGURE 1:**
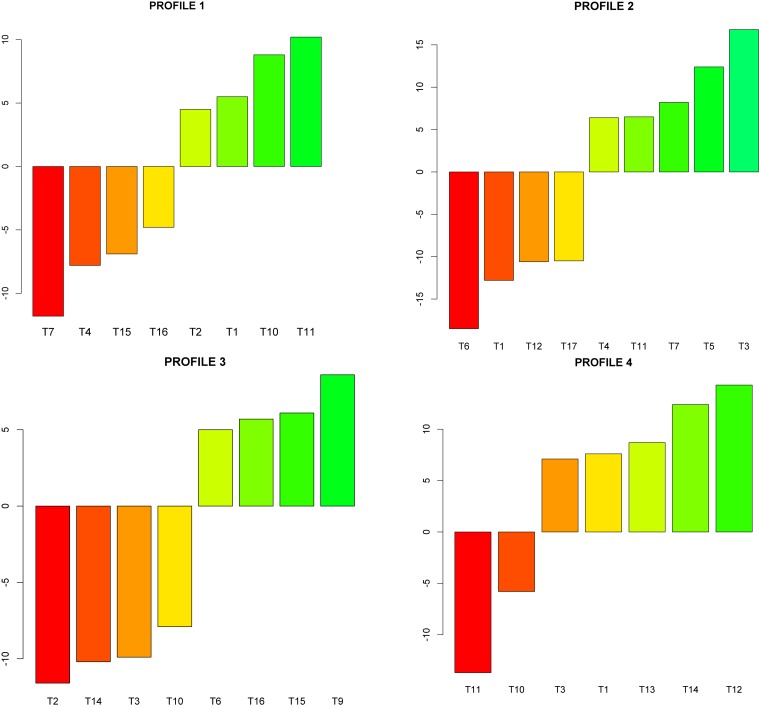
Temperament profiles.

For the interpretation of profiles, we regressed all temperament scales on each one of the 4 profiles selected with LPA. We used cross-validation to find the optimal number of components. Finally, we extracted all the useful information and the output was formatted to obtain normalized regression coefficients (positive and negative) to be interpreted as percentages (so their absolute sum is 100) and the result was sorted. Figure 1 summarizes this process and details the relative importance of the main temperament scales, identifying each profile.

Profile 1 represented 25.8% of the sample (48 boys and 28 girls) and was characterized by higher than average scores in T1, T2, T10, and T11, and well below the mean in T4, T7, T15, and T16. Thus, we labeled this profile as “Outgoing/Average regulation.” Profile 2 comprised 19.3% of the sample (33 boys and 24 girls) and was well above the mean in T4 and T11, higher than the mean in T5, T7, T9, T10, and T15, well below the mean in T6 and lower than the mean in T2, T12 and T17. This profile was then referred to as “Emotionally negative/Low regulation.” Profile 3 represented 23.7% of the sample (36 boys and 34 girls) and was characterized by scores well above the mean in T9, higher than the mean in T6, T16, and T15 and well below the mean in T2, T3, T10, and T14. We called this profile “Inhibited/Average regulation.” Finally, Profile 4 contained 31.2% of the sample (33 boys and 59 girls), and it was characterized by scores well above the mean in T12 and T14, higher than the mean in T3, T1, and T13, lower than the mean in T10 and well below the mean in T11. Thus, this profile was labeled as “Sociable/High regulation.”

Profile differences in social and academic adjustment were carried out using planned comparisons ANOVA. Table 3 shows the means of four measures by profiles and gender. Significant profiles effects were found in the explanation of Acceptance, Rejection, Language and Math achievement, in particular comparing profiles 2 and 4 with other profiles. Specifically, those belonging to Profile 2 have a higher probability of being rejected [*F*(1,293) = 6.65, *p* = 0.01] a lower probability of being popular [*F*(1,293) = 6.51, *p* = 0.01] and score lower in Language [*F*(1,293) = 9.06, *p* = 0.003], and Math achievement [*F*(1,293) = 7.38, *p* = 0.007] than children with other profiles. The inverse pattern was observed in Profile 4. Thus, children belonging to this profile scored significantly lower in Rejection scores: [*F*(1,293) = 21.90, *p* < 0.001], and significantly higher in Acceptance [*F*(1,293) = 15.63, *p* < 0.001], Language [*F*(1,293) = 12.87, *p* < 0.001] and Math performance [*F*(1,293) = 7.28, *p* = 0.007] than children with other profiles.

**TABLE 3:** Means of main measures by profiles and gender.

Measures	Gender	Profile 1	Profile 2	Profile 3	Profile 4
Acceptance	Boys	5.250	4.152	6.611	8.212
	Girls	6.920	5.271	6.441	8.220
Rejection	Boys	7.875	9.394	5.417	3.424
	Girls	5.179	5.283	4.735	1.966
Language	Boys	3.042	2.818	3.222	3.485
	Girls	3.286	3.202	3.412	3.458
Mathematics	Boys	3.145	3.000	3.278	3.576
	Girls	3.143	3.125	3.412	3.322

Finally, after comparing profiles 2 and 4 with gender variable added, we also found significant gender differences on Rejection: *F*(1,292) = 8.52, *p* < 0.01, with boys being more probably rejected, and Language: *F*(1,292) = 4.65, *p* < 0.05, with girls scoring higher in language performance.

## Discussion

Our study had a number of aims. First, we sought to determine temperament profiles in a Spanish sample of 6- and 7-year-old children, and second, we sought to explore how belonging to the different profiles affects children’s academic and social adjustment. Regarding our first objective, we used LPA to identify 4 profiles of children differing in their temperament characteristics. Profile 1 (Outgoing/Average regulation) describes approaching and not very shy children. Children included in this profile, though considered by their parents as active and impulsive, scored as average in effortful control dimensions, such as activation control, inhibitory control and attentional focusing; profile 2 (Emotionally negative/Very low regulation) includes children high in negative emotionality dimensions, such as anger, distress, fear and sadness. On the other hand, these children scored very low in self-regulation dimensions, such as activation control, attentional focusing, inhibitory control and soothability. Profile 3 (Inhibited/Average regulation)takes ininhibited, fearful and shy children, with average scores in effortful control scales, and Profile 4 (Sociable/High regulation) describes quiet and sociable children, with high scores in self-regulation dimensions, such as activation control, inhibitory control, low intensity pleasure and perceptual sensitivity. These profiles were conceptually similar in many aspects to others found in literature. For example, Profile 1 is similar to the profiles “Confident” (Caspi and Silva, 1995) “Social/eager to try” (McClowry, 2002), and “Non-reactive/outgoing” (Sanson et al., 2009) describing approaching and confident children. Profile 2 share characteristics with the profiles “Difficult temperament” (Thomas et al., 1968), “Undercontrolled” (Caspi and Silva, 1995), “High Maintenance” (McClowry, 2002) and “Poor Attention regulation” (Sanson et al., 2009), describing emotionality negative and undercontrolled children. Profile 3 is similar to the profiles “Slow to warm up” (Thomas et al., 1968), “Inhibited” (Caspi and Silva, 1995), “Cautious/Slow to warm up” (McClowry, 2002) and “Reactive/Inhibited” (Sanson et al., 2009), describing fearful and inhibited children. Finally, Profile 4 can be assimilated with profiles “Easy temperament” (Thomas et al., 1968), “Well-adjusted” (Caspi and Silva, 1995), “Industrious” (McClowry, 2002) and “High Attention Regulation” (Sanson et al., 2009), describing adjusted and well-regulated children.

As for our second objective, we found significant differences in academic and social outcomes when we compared the different profiles. With respect to Language and Math achievement, we found that Profile 2 (Emotionally negative/Low regulation) and Profile 4 (Sociable/High regulation) explained children’s performance, suggesting that only children who are outstanding for their high or low attentional capabilities are see their academic performance affected positively or negatively. As expected, children belonging to Profile 2 had a higher probability of academic failure, in both Language and Math, while children belonging to Profile 4 showed the opposite. The literature confirms the importance of self-regulation abilities in the classroom, since the focusing and maintaining attention problem puts the children at risk of feeling overwhelmed and, consequently, missing learning opportunities (Zhou et al., 2010; Hernández et al., 2017). In addition, the connection between self-regulation and learning could be particularly meaningful in Spain, where the more rigid and less spontaneous structure, with scarcity of play time, could “punish” less attentive and self-regulated children (Galián et al., 2018). Few studies have analyzed the relations between temperament and academic achievement from a person-centered perspective, but they replicated the line of results found in ours (Robins et al., 1996; Hart et al., 2003).

We also explored the relations between belonging to a particular profile and the probability of being accepted or rejected in the classroom context. As with academic performance, only Profiles 2 (Emotionally negative/Low regulation) and 4 (Sociable/High regulation) explained children’s social outcomes. Concretely, children in Profile 2 showed a higher probability of being rejected, and a lower probability of being accepted by their classmates. In contrast, children belonging to Profile 4 were more popular, with a lower probability of being rejected and a higher probability of being accepted by their peers. Again, children’s self-regulation abilities seem to be crucial in their social adjustment, and the result suggests that being more inhibited or approaching does not determine children’s sociometric status as much as their ability to regulate their emotions in conflicts and social exchanges. Possible explanations as to why children categorized as shy and inhibited do not show a higher probability of being rejected, as occurs in other cultures, are that inhibited children in our study showed average scores in self-regulation and that Spain still shows traits of collectivistic cultures, where shyness is not as punished as in individualistic ones. Other studies that have examined in depth the relations between temperament and social adjustment from a person-centered approach have highlighted the importance of self-regulation in children’s social development (Sanson et al., 2009; Laible et al., 2010).

The educational implications of these results need to be discussed. On the one hand, it is necessary to work on the awareness among educational agents of the importance of knowing the different temperament profiles that children can show and the effects that belonging to different categories could have on their academic and social trajectories. In this line, it is important to detect temperament profiles that are in higher risk of maladaptation, such as Emotionally negative/Low regulation, found in our sample, so the training of self-regulation strategies should be considered at prevention and intervention level. In this respect, some child-temperament program has proved its efficiency in schools (INSIGHTS; McClowry, 2014), but temperamental applications in educational settings in Spain remain unexplored. A deep reflection from the institution is also needed to facilitate the adaptation of “difficult children” to the system by making the structure progressively more flexible. Actions such as introducing breaks between demanding attentional tasks and letting the children move and play in the classroom at some moments could be very beneficial for children with more challenging temperamental profiles.

Some limitations of the present study should be mentioned. First, it would be interesting to include another sources of information, such as laboratory measures, in addition to parents, in order to increase future study’s inter-reliability. Indeed, a longitudinal analysis of relations between temperament and adjustment from a person-centered approach would be useful, if aimed at exploring whether temperament profiles are stable over different developmental stages, and whether their impact on academic and social adjustment changes over time. Finally, a comparison between temperamental profiles from different cultures could contribute to better understanding of how culture idiosyncrasy and temperament work interrelatedly in children’s adjustment.

## Data Availability Statement

The raw data supporting the conclusions of this article will be made available by the authors, without undue reservation, to any qualified researcher.

## Ethics Statement

This study was carried out in accordance with the recommendations of University of Murcia, with written informed consent from all participants in accordance with the Declaration of Helsinki.

## Author Contributions

All authors designed the study, collected the data, contributed to the interpretation of the results, and revised the final manuscript. EA ran the statistical analyses and wrote the manuscript.

## Conflict of Interest

The authors declare that the research was conducted in the absence of any commercial or financial relationships that could be construed as a potential conflict of interest.

## References

[B1] Ato E. Galián M. D. Fernández-Vilar M. A. (2014). Gender as predictor of social rejection: the mediating/moderating role of effortful control and parenting. *Anal. Psicol.* 30 1069–1078.

[B2] Baudry J. P. (2015). Estimation and model selection for model-based clustering with the conditional classification likelihood. *Electron. J. Stat.* 9 1041–1077. 10.1214/15-ejs1026

[B3] Bergman L. R. Magnusson D. (1997). A person-oriented approach in research on developmental psychopathology. *Dev. Pychopathol* 9 291–319. 10.1017/s095457949700206x 9201446

[B4] Biernacki C. Celeux G. Govaert G. (1998). *Assessing a Mixture Model for Clustering with the Integrated Classification Likelihood.* Piscataway, NJ: IEEE.

[B5] Blair C. Razza R. P. (2007). Relating effortful control, executive function, and false belief understanding to emerging math and literacy ability in Kindergarten. *Child Dev.* 78 647–663. 10.1111/j.1467-8624.2007.01019.x 17381795

[B6] Caspi A. Silva P. A. (1995). Temperamental qualities at age three predict personality traits in young adulthood: longitudinal evidence from a birth cohort. *Child Dev.* 66 486–498. 7750379 10.1111/j.1467-8624.1995.tb00885.x

[B7] Cerezo F. (2000). *BULL-S: Test de Evaluación de la Agresividad Entre Escolares.* Madrid: Albor-Cohs.

[B8] Checa P. Abundis-Gutierrez A. (2017)) Parenting and temperament influence on school success in 9–13 year olds. *Front. Psychol.* 8:543. 10.3389/fpsyg.2017.00543 28446886 PMC5388739

[B9] Checa P. Rueda M. R. (2011). Behavioral and brain measures of executive attention and school competence in late childhood. *Dev. Neuropsychol.* 36 1018–1032. 10.1080/87565641.2011.591857 22004022

[B10] Crockett L. J. Moilanen K. L. Raffaeli M. Randall B. A. (2006). Psychological profiles and adolescent adjustment: a person-centered approach. *Dev. Psychopathol.* 18 195–214. 16478559 10.1017/S0954579406060111

[B11] Eisenberg N. Fabes R. A. Guthrie I. K. Reiser M. (2000). Dispositional emotionality and regulation: their role in predicting quality of social functioning. *J. Pers. Soc. Psychol.* 78 136–157. 10.1037/0022-3514.78.1.136 10653511

[B12] Frick P. J. Morris A. S. (2004). Temperament and development pathways to conduct problems. *J. Clin. Child Adolesc. Psychol.* 33 54–68. 10.1207/s15374424jccp3301_6 15028541

[B13] Galián M. D. Ato E. Fernández-Vilar M. A. (2018). Sociometric Status as Mediator in the Relation Between Effortful Control and Achievement. *Merr. Pal. Quart.* 64 309–328. 10.13110/merrpalmquar1982.64.3.0309

[B14] Giancola P. R. Mezzich A. C. Tarter R. E. (1998). Executive cognitive functioning, temperament, and antisocial behavior in conduct-disordered adolescent females. *J. Abnor. Psychol.* 107 629–641. 10.1037/0021-843x.107.4.629 9830250

[B15] Hallquist M. N. Wiley J. F. (2018). Mplus automation: an R package for facilitating large-scale latent variable analysis in Mplus. *Struct. Equat. Model.* 25 621–638. 10.1080/10705511.2017.1402334 PMC6075832 30083048

[B16] Harring J. R. Hodis F. A. (2016). Mixture modeling: applications in educational psychology. *Educ. Psychol.* 51 354–367. 10.1080/00461520.2016.1207176

[B17] Hart D. Atkins R. Fegley S. Robins R. W. Tracy J. L. (2003). Personality and development in childhood: a person-centered approach. *Mon. Soc. Res. Child Dev.* 68 1–109. 12875200

[B18] Hernández M. M. Valiente C. Eisenberg M. Berger R. H. Spinrad T. L. VanSchyndel S. K. (2017). Elementary students′ effortful control and academic achievement: the mediating role of teacher-student relationship quality. *Early Child. Res. Quart.* 40 98–109. 10.1016/j.ecresq.2016.10.004 28684888 PMC5495479

[B19] Hintsanen M. Alatupa S. Jokela M. Lipsanen J. Hintsa T. Leino M. (2012). Associations of temperament traits and mathematics grades in adolescents are dependent on the rater but independent of motivation and cognitive ability. *Learn. Indiv. Differ.* 22 490–497. 10.1016/j.lindif.2012.03.006

[B20] INEE (2015). *PISA Inform About Educational Assessment From Spain Government.* Available at: https://www.educacionyfp.gob.es/inee/evaluaciones-internacionales/pisa/pisa-2015.html (accessed June 25, 2019).

[B21] Laible D. Carlo G. Panfile T. Eye J. Parker J. (2010). Negative emotionality and emotion regulation: A person-centered approach to predicting socioemotional adjustment in young adolescents. *J. Res. Pers.* 44 621–629. 10.1016/j.jrp.2010.08.003

[B22] Leve L. D. Kim H. K. Pears K. C. (2005). Childhood temperament and family environment as predictors of internalizing and externalizing trajectories from ages 5 to 17. *J. Abnor. Child Psychol.* 33 505–520. 10.1007/s10802-005-6734-7 16195947 PMC1468033

[B23] McClowry S. G. (2002). The temperament profiles of school-age children. *J. Pediat. Nurs.* 17 3–10. 10.1053/jpdn.2002.30929 11891489

[B24] McClowry S. G. (2014). *Temperament-Based Elementary Classroom Management.* Lanham, MD Rowman and Littlefield.

[B25] Mevik B. H. Wehrens R. Liland K. H. (2019). *pls: Partial Least Squares and Principal Component Regression. R Package Version* 2.7–1 Available at: https://CRAN.R-project.org/package=pls

[B26] Ministry of Education and training (2019). *Datos y Cifras del Curso Escolar 2018-. 2019.* Available at: https://sede.educacion.gob.es/publiventa/datos-y-cifras-curso-escolar-20182019/ensenanza-estadisticas/22495 (accessed July 12, 2019).

[B27] Moffitt T. E. Caspi A. Rutter M. Silva P. A. (2001). *Sex Differences in Antisocial behaviour: Conduct Disorder, Delinquency, and Violence in the Dunedin Longitudinal Study*. New York, NY:Cambridge University Press.

[B28] Muthén L. K. Muthén B. O. (1998–2017) *Mplus User’s Guide* 8th Edn. Los Angeles, CA Muthén and Muthén

[B29] Report Bullying sin fronteras (2018). Statistics of Bullying from Spain, 2018. Available at https://bullyingsinfronteras.blogspot.com/2017/05/estadisticas-de-bullying-en-espana-mayo.html (accessed June 7, 2019).

[B30] Robins R. W. John O. P. Caspi A. Moffit T. E. Stouthamer-Loeber M. (1996). Resilient, overcontrolled and undercontrolled boys: three personality types in early adolescence. *J. Pers. Soc. Psychol.* 70 157–171. 10.1037/0022-3514.70.1.157 8558407

[B31] Rosenberg J. M. Beymer P. N. Anderson D. J. Schmidt J. A. (2018). tidyLPA: An R package to easily carry out latent profile analysis (LPA) using open-source and commercial software. *J. Open Sourc. Soft.* 3:978 10.21105/joss.00978

[B32] Rosenberg J. M. van Lissa C. J. Beymer P. N. Anderson D. J. Schell M. J. Schmidt J. A. (2019). *tidyLPA 1.0.0: Easily carry out Latent Profile Analysis (LPA) using open-source or commercial software.* Available at: https://data-edu.github.io/tidyLPA/ (accessed July 3, 2019).

[B33] Rothbart M. K. (1981). Measurement of temperament in infancy. *Child Dev.* 52 569–578.

[B34] Rothbart M. K. Bates J. E. (1998). “Temperament,” in *Handbook of Child Psychology: Social, Emotional and Personality Development*, 5th Edn, Vol. 3 eds Damon W. Eisenberg N., (New York, NY: Wiley), 105–176.

[B35] Sánchez-Pérez N. Fuentes L. J. Pina V. López-López J. A. González-Salinas C. (2015). How to different components of effortful control contribute to children′s mathematics achievement? *Front. Psychol.* 6:1383 10.3389/fpsyg.2015.01383 PMC4584978 26441758

[B36] Sanson A. Smart D. Prior M. Toumborou J. W. Oberklaid F. (2009). Associations between early childhood temperament clusters and later psychological adjustment. *Merr. Pal. Quart.* 55 26–54. 10.1353/mpq.0.0015

[B37] Sclove S. (1987). Application of model-selection criteria to some problem in multivariate analysis. *Psychometrika* 52 333–343. 10.1371/journal.pone.0014147 21188141 PMC3004794

[B38] Simonds J. Rothbart M. K. (2004). “The temperament in middle childhood questionnaire (TMCQ),” in *Poster Session Presented at the Occasional Temperament Conference*, Athens, GA.

[B39] Thomas A. Chess S. Birch H. G. (1968). *Temperament and Behavior Disorders in Children.* Oxford: New York U. Press.

[B40] Valiente C. Lemery-Chalfant K. Swanson J. Reiser M. (2008). Prediction of children’s academic competence from their effortful control, relationships and classroom participation. *J. Educ. Psychol.* 100 67–77. 10.1037/0022-0663.100.1.67 21212831 PMC3014585

[B41] Zhou Q. Main A. Wang Y. (2010). The relations of temperament reactivity and effortful control to children’s adjustment problems in China and the United States. *Dev. Psychol.* 45 724–739. 10.1037/a0013776 PMC4080919 19413428

